# Preliminary evidence for human globus pallidus pars interna neurons signaling reward and sensory stimuli

**DOI:** 10.1016/j.neuroscience.2016.04.020

**Published:** 2016-07-22

**Authors:** Nicholas A. Howell, Ian A. Prescott, Andres M. Lozano, Mojgan Hodaie, Valerie Voon, William D. Hutchison

**Affiliations:** a Institute of Medical Science, Faculty of Medicine, University of Toronto, 1 King’s College Circle, Toronto, Ontario M5S1A8, Canada; b Division of Brain Imaging & Behaviour – Systems Neuroscience, Toronto Western Research Institute, University Health Network, 399 Bathurst Street, Toronto, Ontario M5T 2S8, Canada; c Department of Physiology, Faculty of Medicine, University of Toronto, 1 King’s College Circle, Toronto, Ontario M5S 1A8, Canada; d Division of Neurosurgery, Department of Surgery, Krembil Neuroscience Center, University of Toronto, 399 Bathurst Street, Toronto, Ontario M5T 2S8, Canada; e Department of Psychiatry, University of Cambridge, Herchel Smith Building for Brain & Mind Sciences, Cambridge CB2 0SZ, UK; f Behavioural and Clinical Neurosciences Institute, University of Cambridge, Sir William Hardy Building, Downing Street, Cambridge CB2 3EB, UK; g Cambridgeshire and Peterborough NHS Foundation Trust, Fulbourn Hospital, Cambridge CB21 5EF, UK

**Keywords:** BG, basal ganglia, CD, cervical dystonia, DBS, deep brain stimulation, l-Dopa, levodopa, GPi, globus pallidus pars interna, PD, Parkinson disease, globus pallidus pars interna, reward, sensory, electrophysiology, basal ganglia, human neuroscience

## Abstract

The globus pallidus pars interna (GPi) is a component of the basal ganglia, a network of subcortical nuclei that process motor, associative, and limbic information. While non-human primate studies have suggested a role for the GPi in non-motor functions, there have been no single-unit studies of non-motor electrophysiological behavior of human GPi neurons. We therefore sought to extend these findings by collecting single-unit recordings from awake patients during functional stereotactic neurosurgery targeting the GPi for deep brain stimulation. To assess cellular responses to non-motor information, patients performed a reward task where virtual money could be won, lost, or neither, depending on their performance while cellular activity was monitored. Changes in the firing rates of isolated GPi neurons after the presentation of reward-related stimuli were compared between different reward contingencies (win, loss, null). We observed neurons that modulated their firing rate significantly to the presentation of reward-related stimuli. We furthermore found neurons that responded to visual-stimuli more broadly. This is the first single-unit evidence of human GPi neurons carrying non-motor information. These results are broadly consistent with previous findings in the animal literature and suggest non-motor information may be represented in the single-unit activity of human GPi neurons.

## Highlights

• Non-motor responses of human GPi neurons are described.
• Cells were identified that showed increased firing to reward-stimuli.
• Visual-sensory responses unrelated to reward also observed.

## Introduction

The basal ganglia (BG) are a collection of subcortical nuclei that process a variety of input related to motor, associative, and limbic functions (Alexander et al., 1986). One component, the globus pallidus pars interna (GPi), represents one of two ’output’ nuclei in the circuit. In non-human primates, the GPi receives input from a variety of sources including the striatum, the subthalamic nucleus and the globus pallidus pars externa and projects primarily to ventral anterior and lateral nuclei of the thalamus, indirectly connecting with cortical sites (Hoover and Strick, 1993, Middleton and Strick, 1994, Middleton and Strick, 2002, Parent and Hazrati, 1995). Autoradiographic connectivity studies have also identified limbic projections from the ventral striatum to the rostromedial GPi as well as projections from the ventral pallidum (Alexander et al., 1986, Haber et al., 1990, Haber and Knutson, 2010). While the motor functions of the GPi have been explored in animal models as well as clinically, where it is a target for deep brain stimulation (DBS) surgeries in Parkinson disease (PD) and dystonia, its non-motor functions are less well understood (Lombardi et al., 2000, Haber and Knutson, 2010, DeLong and Wichmann, 2015).

Several non-human primate studies have demonstrated the responsiveness of GPi neurons to reward information and performance of goal-oriented action (Vidailhet et al., 2005, Hong and Hikosaka, 2008, Joshua et al., 2009, Shin and Sommer, 2010, Bromberg-Martin et al., 2010b, Tachibana and Hikosaka, 2012). In particular, the work of Hikosaka and colleagues has described a reward signaling pathway extending from the GPi to the dopaminergic midbrain (Hong and Hikosaka, 2008, Bromberg-Martin et al., 2010b, Hong et al., 2011). These non-motor GPi neurons sent a phasic burst of action potentials similar to those that have been identified in other areas of the reward system (Apicella et al., 1992, Mirenowicz and Schultz, 1994, Schultz et al., 1997, Schultz, 2007). These electrophysiological and behavioral observations are supported by anatomic studies showing connections from neurons in the GPi to the lateral habenula (Parent, 1979, Parent et al., 2001). By communicating with downstream dopaminergic neurons via the lateral habenula, the GPi may influence reward behavior via nuclei receiving dopaminergic input such as the ventral pallidum, a region of pallidal neurons extending from beneath the anterior commissure to areas of the rostromedial GPi and rostral globus pallidus pars externa, or the subthalamic nucleus, nuclei that have both been associated with reward functions (Haber and Knutson, 2010).

The reward processing functions of the human globus pallidus, however, remain poorly understood. Clinical studies of stroke patients have shown reward- and motivation-related deficits such as anhedonia and apathy arising from damage involving this structure (Bhatia and Marsden, 1994, Miller et al., 2006, Vijayaraghavan et al., 2008, Adam et al., 2013). This evidence, however, is non-specific and does not demonstrate the existence of reward-responsive neurons nor their underlying properties. Neuroimaging and local field potential analyses have shown activation of the globus pallidus associated with reward predictions or outcomes (Bischoff-Grethe et al., 2015, Schroll et al., 2015), and have suggested that functional connections may exist between the GPi and lateral habenula (Ide and Li, 2011) similar to what was observed in non-human primates, though some of the findings have not always been anatomically specific to the GPi or consistently identified within a series of analyses. Single-cell electrophysiological recordings used commonly in non-human primate studies offer unparalleled specificity in understanding neural responses to reward information and would provide a point of comparison with the animal literature. While typically unavailable in humans due to the invasiveness of the recordings, patients undergoing functional neurosurgery targeting the GPi often have microelectrode recordings taken to aid in localizing the structure, providing a unique opportunity to study these neurons (Hutchison and Lozano, 2000). We therefore examined the activity of single neurons in the human GPi to determine whether similar non-motor functions are represented in their electrophysiological activity. Based on previous findings from the non-human primate literature, we hypothesized that we would identify a sub-group of neurons that signaled reward information.

## Experimental procedures

### Participants

Eight patients undergoing functional neurosurgery targeting the GPi were recruited for participation. Four patients were diagnosed with PD, one with multiple systems atrophy, two with cervical dystonia (CD), and one with myoclonus dystonia. Three of the participants were female with an average age of 59.1 ± 9.54 years. For PD patients, the mean levodopa (l-Dopa) equivalent dose of medication (±SD) was 958.75 ± 121.68 mg with Unified Parkinson’s Disease Rating Scale-III OFF/ON (±SD-ON/SD-OFF) of 36.6/19.0 ± 8.94/6.01. Four of eight patients had a recent history of one or more psychiatric co-morbidities (impulse control disorder, social anxiety, depressed mood, obsessive compulsive personality). Surgical inclusion criteria for PD patients are guided by the severity of l-Dopa-induced dyskinesias. Surgeries performed included both pallidotomies and DBS electrode insertion. PD patients underwent an overnight withdrawal of dopaminergic medications (⩾12 h) prior to surgery to allow for testing the effect of stimulation on symptom reversal. Patients included in the study provided written, informed consent, and the experiments were approved by the University Health Network and the University of Toronto Research Ethics Boards.

### Data acquisition

A full review of the procedure for stereotactic functional neurosurgery is provided elsewhere (Hutchison and Lozano, 2000). The protocol for both pallidotomy and DBS electrode (Medtronic Model 3387, Minneapolis, MN, USA) insertion involved targeting the desired structure with magnetic resonance imaging-guided coordinates within a standardized stereotactic space. To do this, stereotactic frames were placed on the patients’ heads under local anesthesia with the final target coordinates at the ventral aspect of the GPi (20 mm lateral from midline, 3–6 mm below the anterior to posterior commissure line, 1–2 mm anterior from the midcommissural point) (Hutchison and Lozano, 2000, Prescott et al., 2014). The twin microelectrodes, enclosed in two connected 23-gauge guide tubes, were then inserted into a cannula and advanced along a linear track continuously recording the local cellular and local field potential activity (Levy et al., 2007). Two hydraulic microdrives were used to manipulate the microelectrodes, limiting the introduction of noise into the recordings. The microelectrodes were constructed of paraylene-C insulated tungsten and were sequentially gold and platinum plated to attain an impedance of 0.2–0.4 MΩ at 1000 Hz. The electrodes were separated by approximately 600–800 μm and each recorded unique cellular activity. As cells were encountered, their electrophysiological characteristics were noted and recorded for use in determining the anatomical localization. After passing through the GPi, the electrodes entered the optic tract. This was confirmed with microstimulation; optic track activation results in patients reporting flashes of light in the contralateral visual field in a region lateral to or near the midline (Vitek et al., 1998).

### Task

**Fig. 1:**
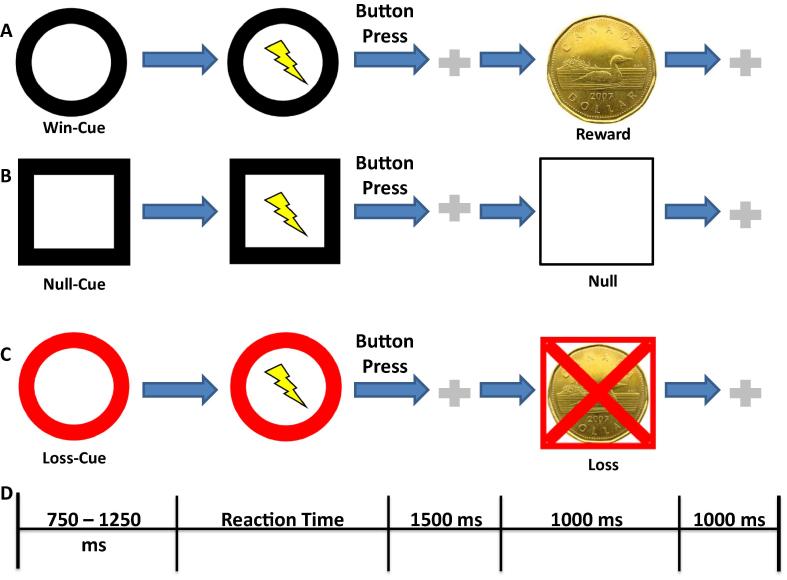
Schematic of the behavioral task. (A) In win trials, a black circle is used as a cue. After the cue is displayed for a variable time, a lightning bolt symbol appears prompting the patient to button press. A fixation point is then displayed, followed by a feedback screen. If the reaction time was small enough, $1 is awarded, indicated by a picture of a one dollar coin (Canadian Dollar). Otherwise, no money is gained or lost, no picture is displayed, and the words “GO FASTER!!” appear. In both cases the net amount of money won or lost is displayed on the screen. (B and C) The process for the no-win and loss trials is the same, except for the differences in cue and outcomes possible. In the case of no-win trials, the outcome screen contains only the patient’s net amount of money for the experiment up to that point. For loss trials, fast reaction times only display net money and slow reaction times additionally display an image of a crossed one-dollar coin with the words “GO FASTER!!” displayed above. (D) The respective duration of each stimulus for one trial. Triggers are created by the software program at the beginning of the cue, lightning bolt, and outcome screen presentations as well as at the time of the button press.

The behavioral task was a modified version of the Monetary Incentive Delay task developed by Knutson and colleagues (Knutson et al., 2000). A schematic diagram of the task is shown in Fig. 1. The task was run on a laptop using E-Prime software (Psychology Software Tools Inc., Sharpsburg, PA, USA). Hereafter, a “trial” will refer to a single sequence of stimulus presentation, patient response, and outcome presentation. A “task” will refer to one full series of trials, including preliminary practice phase and subsequent testing phase, described in detail below. Participants started the experiment with $0 and could ‘win’ or ‘lose’ virtual money based on their performance during the task. Patients were advised that no actual money would be awarded and but that they should attempt to perform as well as possible. A set of instructions was initially displayed that gave examples of each of the stimuli, explained what each symbol meant, and how participants were to complete the task. The patients then performed practice trials that familiarized them with the pace of the experiment and how to respond correctly. All trials in both the practice and testing phases were completed by pressing the designated button as quickly as possible. The participants then began the experiment. Participants completed 3 types of trials: win, null, and loss. The mean reaction time for the practice task was used as a cut-off to which future reaction times were compared. In a win trial, if the patient reacted faster than the cut-off, $1 was awarded. If they failed to do so, no money was won or lost. In null trials, no money was gained or lost regardless of reaction time. In loss trials, patients lost no money if they reacted quickly enough but lost $1 if they did not. Stimuli indicating whether the upcoming trial would be a win, null, or loss trial were displayed for a variable time between 750 and 1250 ms (Fig. 1A–D). This was followed by an image of a lightning bolt, which indicated that they should respond as quickly as possible. Trials could not be advanced until the button press was complete. After the patient’s response, a fixation cross was displayed for 1500 ms, followed by feedback lasting 1000 ms which included whether money was won or lost, the net amount won/lost over the trial to that point, and the words “GO FASTER!!” if the reaction time for the trial was longer than the cut-off. Another fixation cross was displayed for 1000 ms during the inter-trial period. Trial conditions were balanced at 15 win, null, and loss trials each. Three patients were tested using a script that delivered an imbalanced distribution of 27 win trials and 9 trials for null and loss each. All trials were presented in a pseudorandom order.

Markers corresponding to the presentation of each stimulus and response (“triggers”) were recorded throughout the experiment from the laptop running the task (Fig. 1D) and delineated epochs for statistical analysis. Behavioral data related to the task, including reaction time and trial-outcome data, were also saved on the testing laptop for further analysis.

Single-unit microelectrode recordings were collected from GPi neurons during the task. The recordings were amplified, filtered from 100 to 10,000 Hz with two Guideline System GS3000 amplifiers (Axon Instruments, Union City, CA, USA), sampled at 15 kHz with a CED micro 1401 system (Cambridge Electronic Design, Cambridge, UK), and saved to a computer in the operating room. Accelerometer recordings from the wrist and EMG recordings from the extensor carpi radialis and flexor carpi radialis were also taken on the arm used for performing the task. The microelectrodes were advanced until a stable GPi unit was obtained, at which point the behavioral task began. Behavioral tasks were performed at as many sites as possible, testing each site once. Notes were made as to the anatomical location of each testing site for use in track-reconstruction.

### Data analysis

#### Behavioral analysis

Reaction times were only assessed for tasks where participants completed over 75% of the trials. Participants who completed the task multiple times had their performance averaged and treated as a single case for analysis. For each task, trials 3 standard deviations outside the trial-type average reaction time were considered outliers and discarded. One dystonia patient’s data were lost to a recording error. Data were assessed for Normality using the Shapiro–Wilk test. As the reaction times for null trials were not normally distributed (*W* = 0.79, *p* = 0.03), we analyzed the data using a Friedman test with a within-subject factor of trial condition. *Post-hoc* testing used the Holm correction for multiple comparisons.

#### Electrophysiological analysis

Recordings were digitally band pass filtered off-line from 200 to 3000 Hz using Spike 2 software (Version 7, Cambridge Electronic Design, Cambridge, UK) to better isolate single-unit activity. Unique cells were identified by means of the spike sorting program and visual comparison of the wavemark templates. Unique cells identified in this way were considered separately for analysis. Once cells were isolated, their firing rates were divided into 50-ms bins and averaged over all trials for each condition. Individual trials that were contaminated by noise preventing the identification of spikes were discarded. Cells were considered task-related if their averaged firing rates over two or more consecutive bins were outside 2 standard deviations of baseline activity during at least one epoch. The bins exceeding this threshold were used to define the time period for subsequent analysis. Baseline activity was defined as the average of all bins during the 1-s inter-trial interval. All data undergoing statistical testing were assessed for normality using the Shapiro–Wilk test. Cells with firing rates modulated after the presentation of a trial-onset or after trial-outcome cues were tested using either a one-way ANOVA with task condition (Reward, Loss, Null) as a factor or a Kruskal–Wallis test for data that were significantly non-normal. These cells were further assessed for motor activity by comparing task related bins within 0.5 s of a successful button press to baseline activity using a one-way ANOVA or Kruskal–Wallis test, as appropriate. Cells that displayed similar responses across reward conditions were tested for visual-sensory activity. Firing rates were then pooled based on stimulus type according to the same procedure used for trial-type analysis and compared to baseline activity. The three stimulus types treated for analysis were trial-onset cues (black circle, red circle, black square), movement cues (lightning bolt), and trial-outcome cues ($1 coin, crossed-$1 coin, blank square). These cells were subsequently tested with a one-way ANOVA with stimulus type (Trial-Onset Cue, Movement Cue, Trial-Outcome Cue, Baseline) as a factor, or a Kruskal–Wallis test for non-normal data, to determine whether there were significant differences from baseline activity. In both analyses, cells with significant group differences underwent *post hoc* testing to determine their modulation by each condition. Tukey HSD tests were used after a significant ANOVA. *Post hoc* results from Kruskal–Wallis testing were conducted using Dunn’s tests corrected using the Holm method. All analyses were performed using R (Version 3.2.2, R Foundation, Vienna, Austria) and R Studio (Version 0.99, R Studio Inc., Boston, MA, USA) statistical software.

## Results

### Behavioral results

The mean (SD) reaction times for all patients over win, loss, and null trials were 639.2 ms (421.1), 649.1 ms (371.3), and 575.1 ms (333.1), respectively. Considering only dystonic patients (*n* = 2), the mean values were 287.9 ms, 343.8 ms, 385.5 ms for win, loss, and null trials. Considering all patients, no statistically significant difference in reaction times were observed between reward conditions (*χ*^2^_(2)_ = 0.29, *p* > 0.5).

### Single-unit electrophysiology

**Fig. 2:**
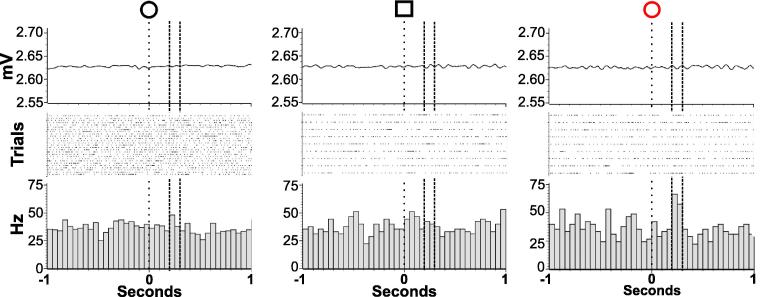
Reward responses of globus pallidus pars interna neurons. Reward-responsive globus pallidus pars interna neuron. The neuron depicted, recorded at +8.0 mm above target, phasically increased its firing rate after presentation of lose (red circle) compared to win trial-onset cues (black circle) (*χ*^2^_(2)_ = 7.97, *p* = 0.02; Win vs. Loss *Z* *=* 2.36, *p* = 0.02; Null vs. Loss *Z* = −2.67, *p* = 0.01; Win vs. Null *p* > 0.05). Top: averaged accelerometer trace. Bottom: averaged firing rate histograms centered on cue presentation. (For interpretation of the references to colour in this figure legend, the reader is referred to the web version of this article.)

**Fig. 3:**
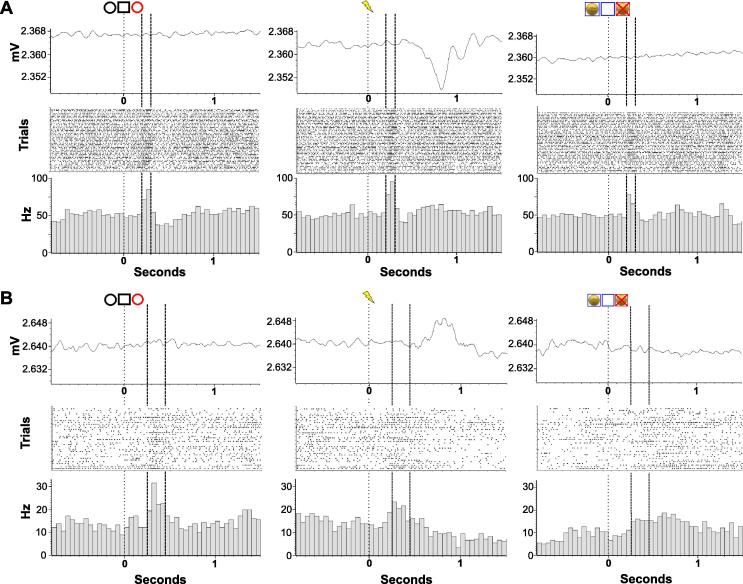
Visual-sensory responses of globus pallidus pars interna neurons. Visual sensory cues elicited responses on similar time scales in GPi cells, although each neuron responded preferentially for a particular type of visual stimulus. The neuron depicted in (A), recorded at +3.4 mm above target, responded significantly more for movement cues compared to trial outcome cues (*χ*^2^_(3)_ = 66.97, *p* < 0.0001; Baseline vs. Onset Cue *Z* = −6.55, *p* < 0.0001; Baseline vs. Movement Cue *Z* = −7.53, *p* < 0.0001; Baseline vs. Outcome Cue *Z* = −4.83, *p* < 0.0001). Responses were often biphasic excitation-inhibitions (e.g. A), but in one instance a phasic excitation (B). The neuron in (B), +2.2 mm above target, indicated a response for onset versus outcome (*χ*^2^_(3)_ = 16.89, *p* < 0.001; Baseline vs. Onset Cue *Z* = −3.68, *p* < 0.001; Baseline vs. Movement Cue *Z* = −2.95, *p* = 0.01, Baseline vs. Outcome Cue *Z* = −1.13, *p* > 0.05; Onset Cue vs. Outcome Cue *Z* = −2.54, *p* = 0.02). Top: averaged accelerometer traces. Bottom: averaged firing rate histograms centered on stimulus presentation.

**Fig. 4:**
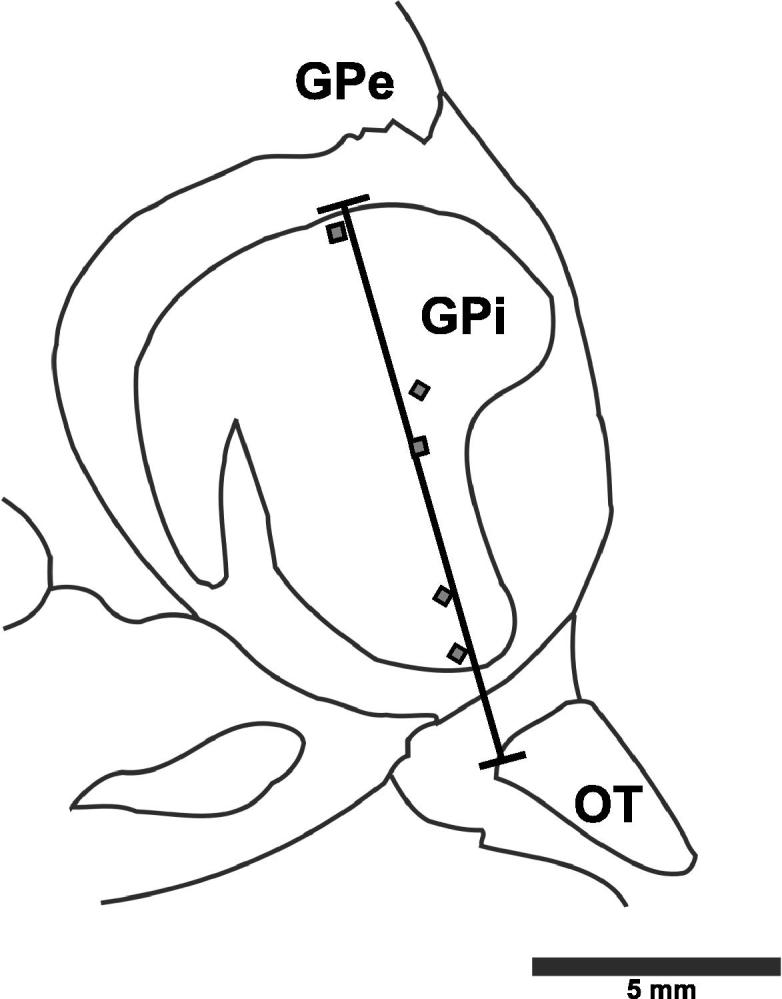
Anatomical localization of task-responsive globus pallidus pars interna neurons. Reconstructed locations of neurons indicating significant responses to reward (square) or visual-sensory (diamond) information. Globus pallidus pars interna (GPi), globus pallidus pars externa (GPe), optic tract (OT). Sagittal section 20.0 mm lateral from midline.

In total eight patients were tested with 20 tasks intra-operatively (range 1–4 tasks/patient) resulting in a bank of 35 cells. Of the GPi cells tested 2 displayed a response to reward stimuli (Fig. 2), and 3 displayed a visual-sensory response (Fig. 3). Fig. 4 shows the locations of neurons observed to have significant responses in the GPi.

#### Reward-valence response

Two GPi cells showed significant modulation by the reward valence condition. One neuron significantly increased its firing rate for loss compared to win cues (*χ*^2^_(2)_ = 7.97, *p* = 0.02; Win vs. Loss *Z* *=* −2.36, *p* = 0.02; Null vs. Loss *Z* = −2.67, *p* = 0.01; Win vs. Null *p* > 0.05) (Fig. 2). The other neuron responded to null cues while being inhibited by loss (*F*(2, 23) = 4.12, *p* = 0.03; Null vs. Loss *p* = 0.02; Null vs. Win & Win vs. Loss *p* > 0.05).

The cells were also described in terms of their cross-modal response. One of the above cells exhibited both motor and reward-sensitive activity. The cell described in Fig. 2 showed a significant phasic inhibition prior to movement (*χ*^2^_(1)_ = 30.49, *p* < 0.001; average movement firing rate = 24.89 Hz, average baseline firing rate = 36.93 Hz) in contrast to its phasic excitation to loss cues. The neurons were drawn from PD and CD patients.

#### Visual-sensory processing

An additional three cells displaying visual-sensory responses were identified incidentally. These neurons were classified based on their robust response to the visual-cues, but lack of discrimination in response between reward-valence conditions. They showed responses in two cases with a biphasic excitation-inhibition pattern (Fig. 3A). The type of visual stimuli eliciting a maximal response, however, differed. One GPi cell had shown a biphasic response to all visual stimuli, but the levels of response were significantly different between categories (*χ*^2^_(3)_ = 66.97, *p* < 0.0001; Baseline vs. Onset Cue *Z* = −6.55, *p* < 0.0001; Baseline vs. Movement Cue *Z* = −7.53, *p* < 0.0001; Baseline vs. Outcome Cue *Z* = −4.83, *p* < 0.0001) (Fig. 3A). *Post hoc* tests revealed that firing to the movement cues was significantly greater than that for the outcome *(Z* = 2.72*, p* = 0.01) with a trend toward increased firing to onset relative to outcome cues *(Z* = 1.76*, p* = 0.08). Another neuron showed a trend toward a response to trial outcomes irrespective of valence, but no significant modulation by trial onset-cues (*χ*^2^_(3)_ = 22.57, *p* < 0.001; Baseline vs. Onset Cue *Z* = 0.78, *p* > 0.05; Baseline vs. Movement Cue *Z* = 2.70, *p* = 0.01; Baseline vs. Outcome Cue *Z* = −1.98, *p* = 0.07; Outcome vs. Onset Cue *Z* = 2.76, *p* = 0.02; Outcome vs. Movement Cue *Z* = 4.69, *p* < 0.0001, Outcome vs. Onset Cue *Z* = −1.91, *p* = 0.06). In contrast, the remaining cell was observed to respond in a selectively enhanced fashion to onset compared to outcome cues with no inhibitory after effect (*χ*^2^_(3)_ = 16.89, *p* < 0.001; Baseline vs. Onset Cue *Z* = −3.68, *p* < 0.001; Baseline vs. Movement Cue *Z* = −2.95, *p* = 0.01, Baseline vs. Outcome Cue *Z* = −1.13, *p* > 0.05; Onset Cue vs. Outcome Cue *Z* = 2.54, *p* = 0.02) (Fig. 3B). Two visual sensory neurons were recorded from a PD patient, while one was recorded from a CD patient.

## Discussion

The GPi serves many distinct functions, encompassing roles in motor, associative, and limbic functioning. Although much is known about the role of motor functioning of the GPi, recent pre-clinical evidence has highlighted reward-related behaviors. The present study offers preliminary evidence suggesting that negative reward signals may also be carried in single human GPi neurons. The reward responses shown were phasic in nature, similar to that reported by Hong and Hikosaka (2008) (Fig. 2). Non-human primate studies suggest that GPi neurons carrying information about negative-rewards are more common than those carrying positive reward signals, which would be consistent with the absence of neurons increasing their firing rate to positive reward in the current sample (Hong and Hikosaka, 2008). This neuron was isolated from an individual with dystonia not suffering from a psychiatric co-morbidity, arguing against its response being confounded by an underlying pathology. Previous reports have emphasized that reward neurons will increase or decrease their firing rate dependent on the reward contingency of the trial (Hong and Hikosaka, 2008). Notably, however, the neurons observed here did not exhibit bidirectional firing changes. Our finding may be a result of the positive stimuli not acquiring equivalent and opposing motivational significance for each participant. This is particularly relevant for PD patients undergoing overnight withdrawal of dopaminergic medications. It may also be more generally related to the nature of the testing environment. Data collection occurred in a context that might reasonably be expected to carry significant stress, potentially affecting the results. Comparison data from primates are also gathered under experimental preparations not replicable in humans, where the liquid rewards commonly used are often delivered after a period of food or water deprivation. It cannot be ruled out, however, that these responses tracked a distinct feature of the trial such as salience. Altogether, while some similarities are observed between the current cells and previous reports, limitations imposed by the testing environment leave uncertainty as to how comparable the electrophysiological responses are between humans and primates.

The number of neurons with significant responses was lower than expected (2/35; 5.7%), and considerably lower than the proportion of cells responding to movement (approximately 35%), although there are several reasons this may have occurred (Hutchison et al., 2003). As noted in the non-human primate literature, the overall population of lateral habenula-projecting GPi neurons is approximately 10% of the structure and previous estimates have found about two-thirds of these neurons respond to reward stimuli (∼7%), comparable to the proportion observed here (Parent, 1979, Parent et al., 2001, Hong and Hikosaka, 2008). These neurons are also preferentially distributed to the rostral pole, the peripheries of the structure, and the area surrounding the accessory medullary lamina (Parent et al., 2001). Given the constraints on data collection posed by the surgical approach, we could not preferentially target regions with higher proportions of reward-responsive neurons, potentially explaining the lower proportion observed here. The surgical environment furthermore limited the number of trials we could perform, lowering our power to detect reward-signals. There are also likely inter-species differences in these cell populations making exact numerical comparisons challenging. In sum, while the results seen here are broadly consistent with those reported in the animal literature, a more robust confirmation of these responses is needed.

The functional significance of GPi reward neurons is still unknown. Clinically, psychiatric syndromes related to globus pallidus lesions include disorders of motivated behavior, such as abulia, a motivational disorder involving a decline in self-generated emotion, actions, and thoughts (Bhatia and Marsden, 1994, Vijayaraghavan et al., 2002, Pagonabarraga et al., 2015). Some results from preclinical and patient research suggest that the GPi may serve to integrate action and motivation. In non-human primates, several studies have shown that cells active during movement can additionally signal reward information or have their activity during movement modulated by reward information, which have been interpreted as representing a ‘binding’ of reward signals to actions (Gdowski et al., 2001, Pasquereau et al., 2007, Shin and Sommer, 2010, Turner and Desmurget, 2010). While primarily interested in the ventral pallidum, Tachibana and Hikosaka (2012) also found that in several instances, inactivation of the GPi with muscimol resulted in alterations of reward-related behaviors. Among patients, case reports have associated abulia and anhedonia with unilateral and bilateral lesions to the globus pallidus (Strub, 1989, Bhatia and Marsden, 1994, Scott et al., 2002, Miller et al., 2006, Vijayaraghavan et al., 2008, Adam et al., 2013). They have not generally, however, commented on whether damage was specific to the GPi. Together these results may support the involvement of the GPi in interfacing between motor and motivational aspects of actions, with lesions potentially manifesting through disorders typified by a flattening of behavioral response to reward and motivational stimuli.

Given work showing the connection of some GPi reward neurons, through the lateral habenula and rostromedial tegmental nucleus, to the dopaminergic midbrain, it is also possible that these neurons affect motivation through their effect on downstream dopamine signaling (Hong and Hikosaka, 2008, Bromberg-Martin et al., 2010a, Bromberg-Martin et al., 2010b, Hong et al., 2011). Dopamine has been linked to the ‘wanting’ aspects of reward, driving consumption behavior (Berridge et al., 2009). The lateral habenula appears to be linked to dopamine neurons that signal motivational value signals (Bromberg-Martin et al., 2010a), which may affect voluntary behaviors through by targeting the rostral caudate (Kim and Hikosaka, 2015). Apathy, which is commonly observed in the context of PD and is sometimes treated with dopaminergic agents, may be a manifestation of dopamine dysregulation (Jorge et al., 2010, Pagonabarraga et al., 2015). Interestingly, in a case report of apathy following bilateral lesions of the GPi, both clinically and experimentally observed deficits in motivation and reward-related behavior were reversed after administration of dopaminergic medications (Adam et al., 2013). Overall, it is possible, although speculative, that lesions of the GPi causing dysregulation of dopamine signaling may then underlie the psychiatric sequelae observed.

While this work might suggest that the reward neurons in the GPi subserve motivational functions, interpretation of these results is challenging. For instance, several distinct subtypes of reward-responsive neurons may exist in the GPi. Neurons exhibiting reward-only and multi-modal responses have both been identified which may be present at different points in the execution of reward-related behaviors (Hong and Hikosaka, 2008, Joshua et al., 2009, Shin and Sommer, 2010). Differences in reward-neurons’ electrophysiological properties have also been observed. Within Hong and Hikosaka’s (2008) study, the neurons carrying negative-reward signals were characterized as ‘border neurons’ with lower firing rates. Conversely, Joshua and colleagues (2009) found GPi cells responsive to reward after explicitly excluding all border cells. It is possible that only some of these groups are responsible for the clinical deficits seen. Future work linking distinct subpopulations of neurons to specific behaviors in animal models of disease may help illuminate these relationships.

Analysis of recordings also revealed neurons with broad responses to stimuli without respect to their reward association*,* potentially reflecting the encoding of visual stimuli. While research on the GPi has not focused on its role in visual processing, primate electrophysiological studies have observed GPi neurons encoding visual stimuli (Aldridge et al., 1980, Shin and Sommer, 2010, Arimura et al., 2013). Visual activity has also previously been reported in multiunit human recordings (Bechtereva et al., 1989). We show that three neurons modulated their activity consistent with visual-sensory stimulation. It should be noted that this task was not designed specifically to test for visual-sensory effects, that these findings were incidental, and that the attribution of these neurons’ activity to visual-sensory processing is provisional. The function of this activity and its recipients are unclear, but may relate to the oculomotor or associative functions of the GPi. Previous work has suggested that GPi neurons encode sequential motor activities in visually guided tasks (Mushiake and Strick, 1995). These visual responses, therefore, may be combined with motor activity to coordinate complex tasks. The differential changes in firing rate observed between stimulus types are consistent with an associative interpretation of these responses. Recent work has also highlighted the role of the GPi in signaling object features that may in turn be used to identify goal-related visual stimuli (Arimura et al., 2013, Hoshi, 2013). It is also possible that the observed activity related to oculomotor movements. While the task did not involve making saccades and all task stimuli were presented centrally, presumably not requiring shifts of gaze, a motor component cannot be ruled out. As in the case of reward-responsive neurons, we identified few visual-sensory neurons in the GPi. Studies in non-human primates, however, have previously found low rates of GPi cells responsive to visual stimuli (1/101; Shin and Sommer, 2010). Nevertheless, further studies offering more robust confirmation of visual-sensory neurons through design and number of neurons recorded would enhance confidence in this finding.

Conducting experiments within patient populations carries a number of inherent limitations which affect the present study. Working in the context of a pathology raises concerns that the findings obtained here may not reflect processes that occur in healthy individuals but are rather reflections of the pathology itself. Furthermore, psychiatric co-morbidities such as depression, frequently encountered in the context of PD (Reijnders et al., 2008, Aarsland et al., 2012), may have affected the reward sensitivity of the participants (Eshel and Roiser, 2010). While this is a distinct possibility, it is currently impossible to obtain similar information from healthy volunteers to address this matter fully. Functional neuroimaging has provided opportunities to investigate changes in neural activity, but it lacks the temporal and spatial resolution provided by electrophysiological techniques. Characteristics such as phasic or bidirectional responses within subpopulations of BG neurons may be lost when averaged through the hemodynamic response. Moreover, in cases such as the GPi, where in this case as few as 7% of neurons in a widely distributed arrangement have been reported to be responsive, attempting to detect this signal can be challenging if not impossible. The intra-operative testing environment is also not ideal for assessing reward-related behaviors. The stress of the ongoing operation, the unfamiliar environment, and the fatigue of the participants’ early morning preparations for the procedure could all contribute to potentially different reward responses compared to what might be achieved in a more relaxed setting. We report here limited responses only to null and lose signals, not rewarding ones. This may in part be caused by the relative devaluation of the reward stimuli by the aforementioned circumstances. As a result of the overnight withdrawal of dopaminergic medications, the motor abilities of many individuals in our sample were also strongly affected, likely influencing our behavioral analyses. While these limitations must be acknowledged, this approach was necessary to investigate this neuronal subpopulation at the required level of specificity.

## Conclusion

In conclusion, we present here preliminary findings from human single neurons suggesting that non-motor information may be carried in the human GPi. We found instances of selective phasic increases in firing rate associated with reward information. We additionally identified neurons that appear to be responsive to visual stimuli. Future studies should target the GPi to confirm these findings and further assess the types and spatial distribution of reward-responses neurons. To address the potential effect of underlying pathology, future work will expand to assess how these responses might vary between patient populations and under differing dopaminergic states. If confirmed, these reward signals may form part of a ’loss information’ pathway described in the animal literature and further support the involvement of the human GPi in non-motor information processing.
